# Expression of p53 as a biomarker in determining response to apatinib for advanced gastric cancer

**DOI:** 10.3389/fonc.2023.1203980

**Published:** 2023-08-15

**Authors:** Zhiyuan Qiu, Rong Qin, Ziyi Zhang, Ting Zhang, Zhao Zhang, Chunyue Qiao, Yan Xi, Guangyu Tian, Yan Wang

**Affiliations:** ^1^ Department of Oncology, The Affiliated People’s Hospital of Jiangsu University, Zhenjiang, Jiangsu, China; ^2^ Department of Geriatrics, The Affiliated People’s Hospital of Jiangsu University, Zhenjiang, Jiangsu, China; ^3^ Department of Oncology, Jiangdu People’s Hospital Affiliated to Medical College of Yangzhou University, Yangzhou, Jiangsu, China

**Keywords:** Apatinib, gastric cancer, targeted therapy, p53, Ki67

## Abstract

**Background:**

Apatinib has shown outstanding value in the treatment of advanced gastric cancer (AGC). However, no biomarkers are available to select AGC patients who will benefit from apatinib. The aim of the present study was to investigate the association between p53 and Ki67 expression of and the outcome in AGC patients treated with apatinib.

**Methods:**

From December 2015 to December 2020, 92 AGC patients were enrolled and was retrospectively evaluated. They were given apatinib at a daily dose of 500 or 250 mg every 4 weeks to monitor clinical efficacy and adverse events (AEs). Kaplan-Meier method was used for survival analysis. Expression of p53 and Ki67 was detected by immunohistochemistry (IHC) and correlated with survival.

**Results:**

Among 92 evaluable patients, the objective response rate (ORR) and disease control rate (DCR) were 17.4% and 79.3%, respectively, and none of them achieved a CR, 16 achieved a PR (17.4%) (95% CI 9.8%-26.1%). Stable disease (SD) was observed in 57.6% of patients (95% CI 49.2%-69.9%) and PD in 21.7% of patients (95% CI 13.6%-31.3%). The median progression free survival (mPFS) was 122.7 ± 8.2 days, and the median overall survival (mOS) was 203.4 ± 11.9 days. P53 expression was observed in 35 patients (38.0%) and high expression of Ki67 was detected in 34 patients (37.0%). There was a statistically significant inverse relationship between p53 and Ki67 expression (*P*=0.014). Moreover, p53 was significantly correlated with the OS (*P*=0.018), but Ki67 had no significant influence on OS.

**Conclusions:**

Apatinib showed promising efficiency and was well tolerated as a second-line treatment for AGC patients. AGC patients with p53-negative were likely to benefit from apatinib treatment; however, the expression of Ki67 proteins has no significant impact on the outcome of AGC patients.

## Introduction

Gastric cancer (GC) is a main cause of cancer-related death worldwide, with a particularly high prevalence in China (1, 2). Because GC patients are often in the advanced stages at diagnosis, the survival of GC is usually less than 6 months (3). Surgery and chemotherapy are the main treatment for GC, but the majority of patients will suffer a relapse. Therefore, targeted therapy has been investigated. Apatinib, an oral small-molecule targeted drug, has potential antiangiogenic effects by blocking the intracellular ATP-binding site of VEGFR-2. Preliminary data suggested that apatinib combined with chemotherapy showed promising efficiency as the second line therapy for AGC patients (4).

However, GC is a heterogeneous disease with different subtypes and not all GC patients will benefit from apatinib. For some GC patients, it can prolong their survival time, but for some others, it may have poor efficacy. Therefore, identification of biomarkers that predict the efficacy of apatinib is imperative for deciding whether to use it or not. However, there are no well-established biomarkers for selecting GC patients who will benefit from apatinib.

Some biomarkers are helpful for a better understanding of tumour behaviour. Among the biomarkers, considerable attention has been paid to the p53 and Ki67. The expression of p53 and Ki67 have been demonstrated in some studies to have potential value in identifying the patients who will benefit from chemotherapy (5, 6). It is also possible that the expression of p53 and Ki67 may identify the patients who will benefit from apatinib. In this study, we test the hypothesis that the responses to apatinib might differ depending on the expression status of p53 and Ki67.

## Methods

### Patients

Between December 2015 and December 2020, 92 AGC patients who failed the first-line chemotherapy were enrolled and followed up retrospectively in the Affiliated People’s Hospital of Jiangsu University. Inclusion criteria were set as follows: (1) age ≧̸18 years; (2) pathologically confirmed GC; (3) an Eastern Cooperative Oncology Group performance status (ECOG PS) of 0-2; (4) at least one measurable lesion as defined by Response Evaluation Criteria In Solid Tumors (RECIST) version 1.0; (5) sufficient organ function. Exclusion criteria were set as follows: (1) patients had any one of other primary tumors; (2) diagnosis of central nervous system metastases; (3) patients were in uncontrolled medical conditions (e.g., bleeding tendency); (4) patients were pregnant or breast-feeding. The study was approved by the Ethics Committee of the Affiliated People’s Hospital of Jiangsu University and conducted according to the Declaration of Helsinki. All patients provided written informed consent prior to participation.

### Immunohistochemistry (IHC) analysis

The expression of p53 and Ki67 was detected in the pretreatment GC specimens which were collected from the department of pathology. Immunohistochemical staining was performed using the EnVision System (Dako Cytomation, Carpinteria, CA, USA). The GC specimens were fixed in formalin and sectioned (4 μm sections). Firstly, the sections were dewaxed in xylene and rehydrated in alcohol, followed by antigen retrieval using EDTA for 5 minutes and endogenous peroxidase blocking by incubation in 3% H_2_O_2_ for 15 minutes. Secondly, the sections were incubated with the antibodies of p53 (clone DO-7, Dako Cytomation, Glostrup, Denmark; 1:50) and Ki67 (clone MIB-1, Dako Cytomation; 1:50), then incubated overnight at 4°C, followed by secondary antibody treatment at room temperature for 2 hours and incubation with the streptavidin-horseradish peroxidase complex. Thirdly, 3,3-diaminobenzidine (DAB) (Sigma-Aldrich, Germany) and HRP-conjugated anti-rabbit immunoglobulin G (IgG) were used for visualisation, and the sections were counterstained with 1% haematoxylin and imaged by an Olympus light microscope. Appropriate positive controls were included in the immunohistochemical staining and phosphate buffered saline (PBS) was used as a negative control.

The p53 expression was defined as positive when staining more than 10% of cancer cell nuclei; tumors with no staining or less than 10% were defined as negative (7, 8). The percentage of cells with Ki67 expression was calculated from the number of malignant cells in the highest labelled field under high magnification (400×). The median percentage (50%) of Ki67 expression was used as the cut-off; thus, cases above the median value (≥50%) were defined as high Ki67 expression, and cases below the median value (< 50%) were defined as low Ki67 expression. The IHC results were judged by two independent pathologists who were blinded to the clinicopathological information.

### Treatment

Apatinib (SFDA approval no. H20140105) were obtained from Jiangsu Hengrui Medicine Co., Ltd. (Lianyungang, China). Patients received oral apatinib (250 mg to 500 mg once daily) half an hour after a meal, according to the daily medication schedule. Each cycle lasted 28 days and a treatment course consisted of 2 cycles. Treatment was continued until disease progression or unacceptable toxicity. Before treatment, patients underwent a physical examination, assessment of bleeding risk, liver and kidney function tests, routine blood tests and computed tomography (CT) scans.

### Efficacy and safety

The primary endpoints were progression free survival (PFS) and overall survival (OS), and the secondary endpoints were objective response rate (ORR) and disease control rate (DCR). PFS was defined from the date of initial treatment to the date of tumor progression or death from any cause. OS was calculated from the date of initial treatment until death or the last follow-up. The clinical efficacy was assessed according to the RECIST version 1.1 and was classified as a complete response (CR), partial response (PR), stable disease (SD), or progressive disease (PD). The ORR was defined as the percentage of patients with a CR or PR, and the DCR was defined as the percentage of patients with a CR, PR, or SD. Adverse events (AEs) were evaluated according to National Cancer Institute Common Terminology Criteria for Adverse Events (NCI-CTCAE) version 4.0.

### Follow-up and outcome

Follow-up by telephone call or clinic consultation was conducted at least once per week. The following items were recorded during follow-up: the disease course, symptoms, AEs, subsequent treatment after PD, status at the end of follow-up (withdrawal, death or survival) and survival time.

### Statistical analysis

All statistical analyses were performed by using SPSS (version 22.0; IBM Corporation, Armonk, NY, USA). Chi-squared test and the Student’s t-test were used for comparisons between two groups. Survivals were calculated using the Kaplan-Meier method and compared using the log-rank test. Cox proportional hazards analysis was used to explore the effects of clinical variables, p53 and Ki67 expression on survival. A two-sided *P*<0.05 was considered statistically significant.

## Results

### Patient characteristics

A total of 92 AGC patients were enrolled into this study, including 62 men (67.4%) and 30 women (32.6%), with a median age of 62.9 ± 8.7 years (range: 30-82). Details of patient characteristics were shown in **Table 1**. All patients received a first-line chemotherapy. Most patients had a favourable ECOG PS (PS 0: 65, 70.7%; PS 1: 19, 20.7%, PS 2: 8, 8.7%). Prior surgery had been performed in 49 patients (53.3%). All patients had metastatic disease, and the predominant metastatic sites were abdominal lymph nodes (75%), liver (41%), and lung (27.2%). Their first-line chemotherapy regimens were as follows: 56% of them received SOX, 21% received docetaxel and S-1, and 2% received S-1 monotherapy (**Table 1**). All patients were evaluated for response, safety and survival.

**Table 1:** 92 AGC patients characteristics.

Characteristics	n	%
Gender		
Male	62	67.4
Female	30	32.6
Median age	62.9±8.7 years (range: 30-82)	
Age (years)		
≤65	56	60.9
>65	36	39.1
ECOG PS		
0	65	70.7
1	19	20.7
2	8	8.7
Metastatic sites, n		
≤2	47	51.1
>2	45	48.9
Sugery of tumor		
no	43	46.7
yes	49	53.3
P53 status		
Negetive	57	62.0
Positive	35	38.0
Ki67 expression		
High	34	37.0
Low	58	63.0

ECOG PS, Eastern Cooperative Oncology Group Performance Status.

### Efficacy and survival analysis

**Figure 1:**
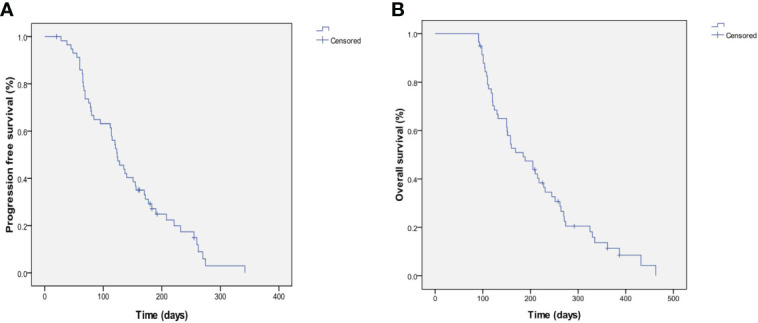
Kaplan-Meier estimates of PFS and OS. **(A)** PFS for the overall population and the median PFS was 122.7±8.2 days (95%CI 106.7-139.0). **(B)** OS for the overall population and the median OS was 203.4±11.9 days (95%CI 180.7-225.5).

At the end of the follow-up, out of a total of 92 patients, 89 patients died from related causes and 3 patients were still alive. Of these, 20 patients received apatinib monotherapy and 72 patients received combination chemotherapy. All patients were evaluated for response, safety and survival (**Table 2**). None achieved a CR, and 16 achieved a PR (17.4%, 95% CI 9.8%-26.1%). Stable disease (SD) was observed in 57.6% (53/92) of patients (95% CI 49.2%-69.9%) and PD in 21.7% (20/92) of patients (95% CI 13.6%-31.3%). The ORR and DCR were 17.4% and 79.3%, respectively. The median PFS (mPFS) was 122.7 ± 8.2 days (95% CI 106.7–139.0), and the median OS (mOS) was 203.4 ± 11.9 days (95% CI 180.7–225.5) (**Figure 1**).

**Table 2:** Overall response.

Response	Patients (n=92)	%
CR	0	0
PR	16	17.4%
SD	53	57.6%
PD	20	21.7%
Missing	3	3.3%
ORR	16	17.4%
DCR	69	79.3%

CI, confidence interval; CR, complete response; DCR, disease control rate; ORR, objective response rate; PD, progressive disease; PR, partial response; SD, stable disease.

### Protein expression by IHC analysis

**Figure 2:**
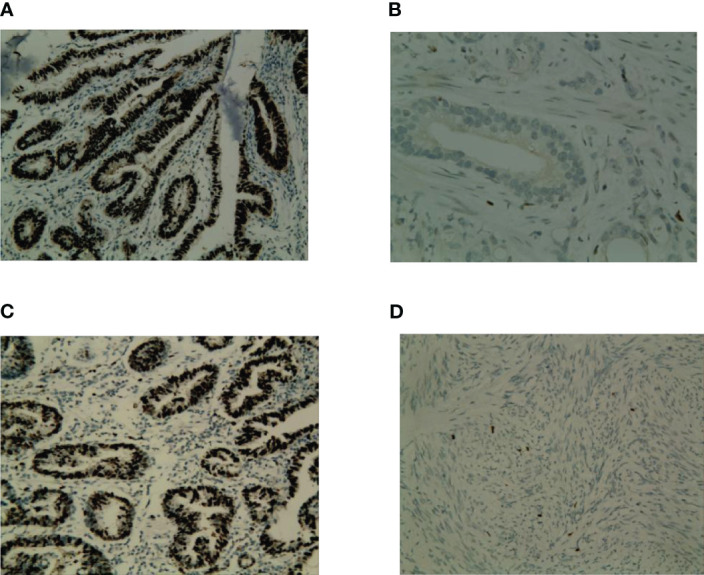
Representative photograps of p53 and Ki67 expression in GC. **(A)** p53-positive expression; **(B)** p53-negative; **(C)** High expression of Ki67; **(D)** low expression of Ki67.

Expression of p53 and Ki67 were evaluated in 92 tumor samples using IHC. Positive staining for p53 was located in the nuclei of tumor cells (**Figures 2A, B**), and positive staining for Ki67 was also located in the nuclei of tumor cells (**Figures 2C, D**). Overall, 38.0% (35/92) of the patients exhibited p53-postive expression, whereas 62.0% (57/92) patients exhibited p53-negative expression. High and low expression of Ki67 were 37.0% (34/92) and 63.0% (58/92), respectively. Ki67 expression was correlated with p53 expression (r=0.42, *P*<0.001). The representative photographs were shown in **Figure 2**.

### The p53 and Ki67 expression and survival

**Figure 3:**
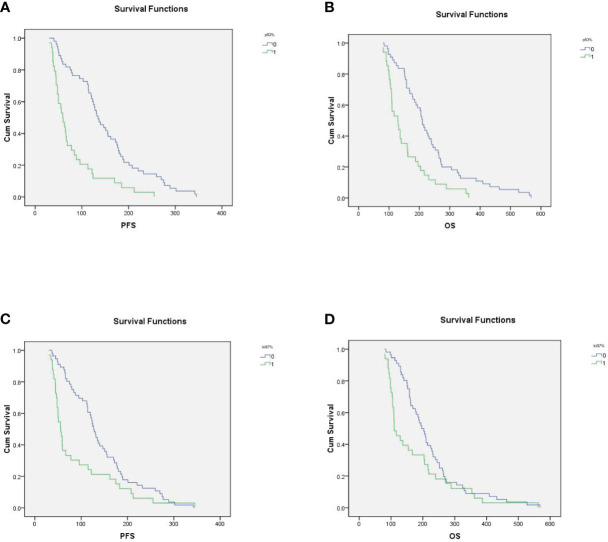
**(A)** In p53-positive and p53-negative patients, mPFS were 78.7 ± 9.2 days and 150.2 ± 10.5 days, respectively (*P<*0.001); **(B)** In p53-positive and p53-negative patients, m0S were 152.5 ± 12.5 days and 234.3 ± 15.4, respectively. **(C)** In high Ki67 expression and low Ki67 expression patients, mPFS were 91.3 ± 13.2 days and 141.4 ± 9.8 days, respectively (*P=*0.011); **(D)** In high Ki67 expression and low Ki67 expression patients, mOS were 172.8 ± 19.5 days and 220.9 ± 13.7 days, respectively (*P*=0.055).

The median PFS and median OS of p53-postive patients were 78.7 ± 9.2 days and 152.5 ± 12.5 days, compared with 150.2 ± 10.5 days and 234.3 ± 15.4 days for p53-negative patients (*P*<0.001, **Figure 3A** and P<0.001, **Figure 3B**). In high Ki67 expression and low Ki67 expression, mPFS were 91.3 ± 13.2 days and 141.4 ± 9.8 days respectively (*P*=0.011, **Figure 3C**); and mOS were 172.8 ± 19.5 days and 220.9 ± 13.7 days respectively (*P*=0.055, **Figure 3D**). In the univariate analysis, ECOG PS and p53-negative were associated with longer OS and PFS. The multivariate analysis showed that ECOG PS and p53-negative were the independent factors of PFS and OS for the AGC patients. Additionally, age, gender and metastatic sites were not risk factors of OS and PFS.

### Safety

All 92 patients were evaluated for AEs. Treatment was generally well tolerated and the treatment-related AEs were mainly manifested as hypertension (46.7%), hand-foot reaction (21.7%), proteinuria (16.3%), fatigue (50%) and hematologic toxicity (15%). The main treatment-related AEs are summarized in **Table 3**. Overall, AEs were moderate and severe episodes were rare.

**Table 3:** Adverse events during the treatment.

Adverse events	Patients (n)	%
Hematologic		
Leukopenia	45	48.9
Anemia	8	8.7
Thrombocytopenia	18	19.6
Nonhematologic		
Fatigue	46	50.0
Hypertension	43	46.7
Proteinuria	15	16.3
Hand-foot syndrome	20	21.7
Diarrhea	6	6.5

## Discussion

AGC patients have poor prognosis, and the main aim of treatment is to alleviate symptoms, improve quality of life, and prolong survival time. Clinical trials have shown that the addition of antiangiogenic drugs to chemotherapy improves the OS in AGC patients compared with chemotherapy alone. In RAINBOW study, the addition of ramucirumab could improve OS in the second-line treatment of AGC patients (9). However, in AVAGAST trial, the addition of bevacizumab to chemotherapy didn’t improve the OS in the first-line treatment of AGC (10). Apatinib has been shown to be effective in the treatment of AGC by inhibiting angiogenesis, decreasing microvascular density of tumor and promoting apoptosis (11). To date, despite years of extensive biomarker research across multiple solid tumour types in patients treated with anti-angiogenic agents, no reproducible predictive biomarkers have been identified. The present retrospective analysis of p53 and Ki67 expression in GC samples reveal a biomarker identifying patients who will benefit from apatinib.

The results of our study indicate that mPFS and mOS were better than previous studies. Lu B et al. reported that the PFS was 3.72 months when apatinib was combined with chemotherapy as the second-line therapy for AGC patients (12). Zhang Y et al. reported that the PFS and OS was 3.06 months and 6.93 months as second-line therapy with apatinib combined with chemotherapy in AGC (13). In another study, the second-line chemotherapy combined with apatinib showed similar efficacy, but the patients had fewer AEs (14). The mPFS in our study was similar with that in RAINBOW study, which was ramucirumab combined with paclitaxel in the second-line setting (9). Therefore, apatinib combined with chemotherapy as the second-line therapy was associated with an increased survival benefit. Treatment was generally well tolerated, and the severity of these AEs was similar to or better than those observed in the previous studies.

GC is a heterogeneous disease, and identifying patients who may benefit from apatinib will help in the selection of appropriate treatment. Without biomarker investigation, the treatment could potentially counteract treatment non-response or drug resistance, but the predictive molecular biomarkers for apatinib have not yet been reported. P53 and Ki67 play crucial roles in the cell proliferation, invasion, angiogenesis and metastasis, and thus may help to define high-risk patients (14). Both p53 and Ki67 have been reported as prognostic biomarkers for survival in GC (15). Thus, we explored the expression status of Ki67 and p53 for their response to apatinib and found the outcome of patients was influenced by p53 expression status.

The p53 gene is “a cellular gatekeeper” and plays a pivotal role in genomic stability. P53 is the most frequently mutated gene and the p53 signaling pathway is frequently perturbed in human cancers (16). P53 mutations gain new oncogenic properties, including deregulated metabolic pathway, increased tumor invasion, inhibition of cell death, and enhanced chemotherapy resistance (16, 17). Normally, wild-type p53 is expressed at low levels, whereas p53 overexpression due to point mutations is frequently detected by IHC (18). Given the difficulties associated with direct sequencing of the p53 gene, we used IHC as a means of detecting mutant p53, with the assumption that p53 mutations result in overexpression of p53 proteins.

P53 was one of the most significant prognostic factors of GC (19, 20). However, the prognostic significance of p53 in GC treatment is limited. Many studies have attempted to correlate p53 expression status with therapeutic response in AGC, but have yielded inconsistent results. It has been demonstrated that p53 expression is a predictive factor for platinum resistance, and platin-free combinations can be used in tumors with p53 overexpression (21, 22). We divided our patients into two subgroups according to the p53 expression status, and 38.0% (35/92) patients showed p53-postive and 62.0% (57/92) patients showed p53-negative. We found that p53-negative patients were associated with longer OS and PFS compared with p53-positive patients. On the basis of our reports, p53 expression is a promising marker that could predict treatment response to apatinib in AGC patients.

Ki67, a downstream effector of p53, plays a key role as a modulator of cell cycle arrest by inhibiting cyclin D1/cyclin-dependent kinase 4/6 (CDK4/6) complexes in G1 to M phases transition. The Ki67 expression, evaluated by IHC, is useful as one of the most reliable markers of the proliferative status of tumour cells. We divided our patients into two subgroups according to the Ki67 expression status. Our results showed that in the patients with low Ki67 expression, the mPFS and mOS were longer than those patients with high Ki67 expression, but only the mPFS was statistically significant. Our results suggest that the effects of apatinib-based chemotherapy in GC patients cannot be predicted by Ki67 alone.

P53 is involved in the cycle of tumor cells and the inactivation of p53 gene contributes to the resistance to anticancer agents. Apatinib could stimulate the expression of apoptotic factors and promote tumor cell apoptosis. Moreover, apatinib can reverse multidrug resistance by inhibiting the function of multiple ABC transporters (23). Apatinib can inhibit the production of VEGF and has a significant effect on the cycle of tumor cells, and then achieves the inhibition of tumor cell proliferation. The combination of apatinib and chemotherapy has synergistic benefits for AGC patients.

However, some limitations should be acknowledged in our study. Firstly, the cohort in our study is relatively small which could result in bias in findings. Therefore, studies with larger sample sizes are needed for verification. Secondly, it was a retrospective study at a single institution. Thirdly, we only used IHC to examine the p53 expression and the p53 mutation was not assessed, so we cannot definitively confirm that we only detected mutant p53 in our IHC analyses. Although next generation sequencing (NGS) allows whole genomic evaluation, it is still impractical to use NGS to determine p53 mutation state due to high cost and complex interpretation. By contrast, IHC is an easy and quick method for detecting p53 mutations. Lastly, the exact mechanisms underlying the involvement of p53 in GC angiogenesis need to be further explored.

In summary, our study indicated that apatinib combined with chemotherapy was both effective and safe as a second-line therapy in AGC patients. Furthermore, we demonstrated that the benefit of apatinib may be more pronounced in patients with p53-negative and p53 can be used in the potential clinic routine to predict the treatment response to apatinib. Providing the accurate pathologic diagnosis, clinicians could make an individualized treatment. To our knowledge, the present study is the first attempt to predict the effects of apatinib using p53 immunostaining in AGC. Our model may facilitate routine prognostic prediction based on the IHC and contribute to the precision oncology, and personalized cancer treatment. However, these results should be confirmed in large-sample, multicenter, randomized controlled prospective studies.

## Data availability statement

The datasets presented in this study can be found in online repositories. The names of the repository/repositories and accession number(s) can be found in the article/supplementary material.

## Ethics statement

The studies involving human participants were reviewed and approved by the Ethics Committee of the Affiliated People’s Hospital of Jiangsu University. The patients/participants provided their written informed consent to participate in this study. Written informed consent was obtained from the individual(s), and minor(s)’ legal guardian/next of kin, for the publication of any potentially identifiable images or data included in this article.

## Author contributions

GT and YW provided direction and guidance throughout the preparation of this manuscript. ZQ and RQ wrote and edited the manuscript. CQ and TZ reviewed and made significant revisions to the manuscript. ZiZ, ZhZ and YX collected and prepared the related papers. All authors contributed to the article and approved the submitted version.

## References

[1] Sung H Ferlay J Siegel RL Laversanne M Soerjomataram I Jemal A. Global cancer statistics 2020: GLOBOCAN estimates of incidence and mortality worldwide for 36 cancers in 185 countries. CA Cancer J Clin (2021) 71(3):209–49. doi: 10.3322/caac.21660 33538338

[2] Chen W Sun K Zheng R Zeng H Zhang S Xia C. Cancer incidence and mortality in China, 2014. Chin J Cancer Res (2018) 30(1):1–12. doi: 10.21147/j.issn.1000-9604.2018.01.01 29545714 PMC5842223

[3] GASTRIC (Global Advanced/Adjuvant Stomach Tumor Research International Collaboration) Group. Role of chemotherapy for advanced/recurrent gastric cancer: an individual-patient-data meta-analysis. Eur J Cancer (2013) 49:1565–77. doi: 10.1016/j.ejca.2012.12.016 23352439

[4] Zhiyuan Q Rong Q Guangyu T Zhao Z Meifang C Han He. Apatinib combined with S-1 as second-line therapy in advanced gastric cancer. Med (Baltimore) (2021) 100(17):e25630. doi: 10.1097/MD.0000000000025630 PMC8084084 33907117

[5] Allegra CJ Paik S Colangelo LH Parr AL Kirsch I Kim G. Prognostic value of thymidylate synthase, Ki-67, and p53 in patients with Dukes’B and C colon cancer: a National Cancer Institute-National Surgical Adjuvant Breast and Bowel Project collaborative study. J Clin Oncol (2003) 21(2):241–50. doi: 10.1200/JCO.2003.05.044 12525515

[6] Popat S Chen Z Zhao D Pan H Hearle N Chandler I. A prospective, blinded analysis of thymidylate synthase and p53 expression as prognostic markers in the adjuvant treatment of colorectal cancer. Ann Oncol (2006) 17(12):1810–7. doi: 10.1093/annonc/mdl301 16971666

[7] Sumiyoshi Y Kakeji Y Egashira A Mizokami K Orita H Maehara Y. Overexpression of hypoxia-inducible factor 1 alpha and p53 is a marker for an unfavorable prognosis in gastric cancer. Clin Cancer Res (2006) 12(17):5112–7. doi: 10.1158/1078-0432.CCR-05-2382 16951228

[8] Goncalves AR Carneiro AJ Martins I de Faria PA Ferreira MA de Mello EL. Prognostic significance of p53 protein expression in early gastric cancer. Pathol Oncol Res (2011) 17(2):349–55. doi: 10.1007/s12253-010-9333-z 21116760

[9] Wilke H Muro K Van Cutsem E Oh SC Bodoky G Shimada Y. Ramucirumab plus paclitaxel versus placebo plus paclitaxel in patients with previously treated advanced gastric or gastro-oesophageal junction adenocarcinoma (RAINBOW): a doubleblind, randomised phase 3 trial. Lancet Oncol (2014) 15:1224–35. doi: 10.1016/S1470-2045(14)70420-6 25240821

[10] Ohtsu A Shah MA Van Cutsem E Rha SY Sawaki A Park SR. Bevacizumab in combination with chemotherapy as first-line therapy in advanced gastric cancer: a randomized, double-blind, placebo-controlled phase III study. J Clin Oncol (2011) 29(30):3968–76. doi: 10.1200/JCO.2011.36.2236 21844504

[11] Yao YW He YF Hu B. Clinical observation of treatment in advanced gastric cancer with apatinib. Chin J Cancer Prev Treat (2017) 24:389–93.

[12] Lu B Lu C Sun Z Qu C Chen J Hua Z. Combination of apatinib mesylate and second-line chemotherapy for treating gastroesophageal junction adenocarcinoma. J Int Med Res (2019) 47(5):2207–14. doi: 10.1177/0300060519827191 PMC6567765 30991863

[13] Zhang Y Xu J Wang Q Ling G Mao Y Cai M. Efficacy and safety of second-line therapy with apatinib combined with chemotherapy as second-line therapy in advanced gastric cancer: a single-arm, open-label, prospective, multicenter study. Ann Transl Med (2022) 10(11):641. doi: 10.21037/atm-22-2752 35813347 PMC9263772

[14] Wang X Zhang R Du N Yang M Zang A Liu L. An open label, multicenter, noninterventional study of apatinib in advanced gastric cancer patients (AHEAD-G202). Ther Adv Med Oncol (2020) 12:1–13. doi: 10.1177/1758835920905424 PMC7082876 32218807

[15] Van Cutsem E de Haas S Kang YK Ohtsu A Tebbutt NC Ming Xu J. Bevacizumab in combination with chemotherapy as first-line therapy in advanced gastric cancer: a biomarker evaluation from the AVAGAST randomized phase III trial. J Clin Oncol (2012) 30(17):2119–27. doi: 10.1200/JCO.2011.39.9824 22565005

[16] Muller PA Vousden KH Norman JC. p53 and its mutants in tumor cell migration and invasion. J Cell Biol (2011) 192(2):209–18. doi: 10.1083/jcb.201009059 PMC3172183 21263025

[17] Muller PA Vousden KH. Mutant p53 in cancer: new functions and therapeutic opportunities. Cancer Cell (2014) 25(3):304–17. doi: 10.1016/j.ccr.2014.01.021 PMC3970583 24651012

[18] Hall PA Lane DP. P53 in tumour pathology: can we trust immunohistochemistry? – Revisited! J Pathol (1994) 172(1):1–4. doi: 10.1002/path.1711720103 7931821

[19] Gabbert HE Muller W Schneiders A Meier S Hommel G. The relationship of p53 expression to the prognosis of 418 patients with gastric carcinoma. Cancer (1995) 76:720–6. doi: 10.1002/1097-0142(19950901)76:5<720::AID-CNCR2820760503>3.0.CO;2-E 8625172

[20] Martin HM Filipe MI Morris RW Lane DP Silvestre F. p53 expression and prognosis in gastric carcinoma. Int J Cancer (1992) 50:859–62. doi: 10.1002/ijc.2910500604 1555884

[21] Lin X Howell SB. DNA mismatch repair and p53 function are major determinants of the rate of development of cisplatin resistance. Mol Cancer Ther (2006) 5(5):1239–47. doi: 10.1158/1535-7163.MCT-05-0491 16731756

[22] Bunz F Hwang PM Torrance C Waldman T Zhang Y Dillehay L. Disruption of p53 in human cancer cells alters the responses to therapeutic agents. J Clin Invest (1999) 104:263–9. doi: 10.1172/JCI6863 PMC408422 10430607

[23] Peng S Zhang Y Peng H Ke Z Xu L Su T. Intracellular autocrine VEGF signaling promotes EBDC cell proliferation, which can be inhibited by apatinib. Cancer Lett (2016) 373:193–202. doi: 10.1016/j.canlet.2016.01.015 26805764

